# Bilateral chylothorax following papillary thyroid carcinoma with cervical lymph node dissection: Case report and comprehensive review of the literature

**DOI:** 10.1097/MD.0000000000040371

**Published:** 2024-11-08

**Authors:** Jing Zhou, Daxue Li, Qian Xiao, Yuchen Zhuang, Ting Yang, Song Xue, Han Gao, Xinliang Su

**Affiliations:** a Department of Breast and Thyroid Department, Women and Children’s Hospital of Chongqing Medical University: Chongqing Health Center for Women and Children, Chongqing, China; b Intelligent Integrated Circuits and Systems Laboratory (SICS Lab), University of Electronic Science and Technology of China, Chengdu, China; c Department of Breast and Thyroid Surgery, The First Affiliated Hospital of Chongqing Medical University, Chongqing, China.

**Keywords:** bilateral chylothorax (BC), chyle leakage (CL), thyroid

## Abstract

**Rationale:**

This case analysis and literature review aim to identify the causes of bilateral chylothorax following thyroid cancer surgery, a rare yet serious complication.

**Patient concerns:**

We report 2 East Asian women who developed bilateral chylothorax after undergoing total thyroidectomy with neck lymph node dissection. Both patients presented with dyspnea and significant pleural effusion postoperatively.

**Diagnoses:**

Both patients were diagnosed with bilateral chylothorax based on clinical examination and imaging studies, including chest ultrasonography and X-rays.

**Interventions:**

In both cases, conservative management was initially implemented, involving chest tube drainage, total parenteral nutrition, and octreotide therapy. Surgical intervention was considered if conservative measures failed to control the chylous output.

**Outcomes:**

Both patients showed gradual improvement with conservative treatment, ultimately resulting in successful resolution of pleural effusion and discharge from the hospital without complications.

**Lessons:**

For patients with bilateral chylothorax, conservative treatment should be the initial approach for small effusions. For moderate to large effusions, placement of a chest drainage tube is recommended, and surgical intervention should be considered if chyle volume exceeds 10 mL/(kg/d) for 48 to 72 hours or persists for more than 11 days following conservative treatment.

## 1. Introduction

The incidence of chylothorax following cervical lymph node dissection (LND) is estimated to be between 2% and 8%.^[1–3]^ However, the occurrence of bilateral chylothorax (BC) following cervical LND is extremely rare. Since Stuart first reported a case of BC after cervical LND in 1907,^[4]^ only a small number of cases, not exceeding 40, have been documented to date. Additionally, the occurrence of BC after surgery for papillary thyroid carcinoma (PTC) is even rarer, with only approximately 20 cases reported in the existing literature.^[5,6]^ The development of postoperative BC after thyroid cancer surgery is believed to be caused by intraoperative damage to the thoracic duct (TD). However, incorrect management of this complication can have severe or life-threatening consequences for the patient.^[7,8]^ The underlying mechanism for the occurrence of BC after CLND for PTC remains unknown. Currently, 2 hypotheses have been proposed to explain the pathophysiological mechanism of BC: the “overflow hypothesis” and the “drain blockage hypothesis.” These hypotheses are still a subject of controversy.^[6,9]^ We believe that a case-by-case analysis is necessary, as the symptoms and signs of chylothorax caused by different mechanisms can present differently and require distinct diagnosis and treatment approaches. Although both cases discussed in this report involve TD injury and treatment, the occurrence and management of BC after surgery vary considerably. By conducting a comprehensive literature review and analysis to understand the mechanisms underlying BC formation, we aim to identify and diagnose BC as early as possible and initiate timely management of chylothorax with minimal harm and discomfort. This approach will help avoid delayed diagnosis and inappropriate treatment, which can lead to serious consequences.

## 2. Case report

This is a retrospective study that reviewed the clinical and pathological records of 2 patients who visited the Affiliated Hospital of Chongqing Medical University Women and Children’s Hospital during September to November 2022. All information on the 2 patients was obtained with their informed consent.

### 2.1. Case 1

**Figure 1.**
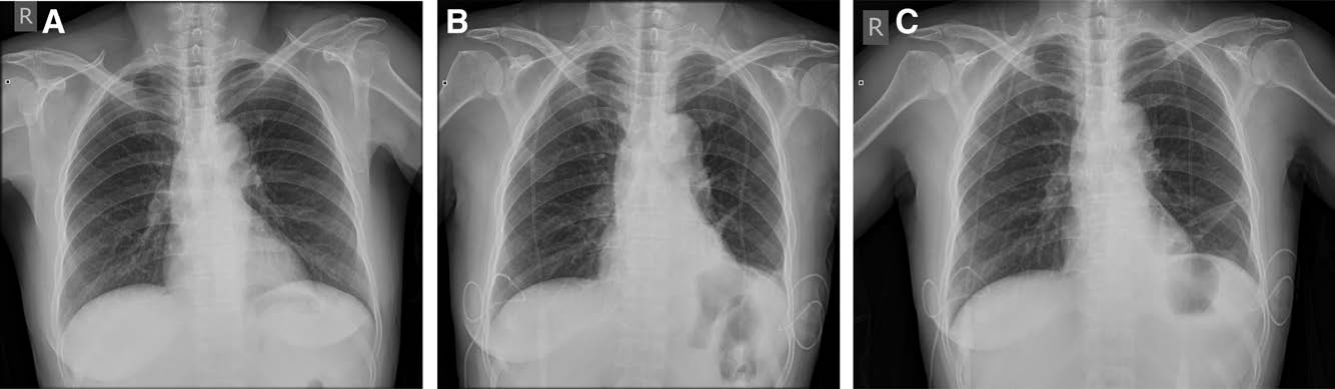
Chest X-rays of case 1: (A) preoperative chest X-ray was normal; (B) postoperative day 4 (POD4) chest X-ray showed lung re-expansion, complete re-expansion of the right lung, and indistinct left costophrenic angle, with bilateral pigtail catheters placed (arrows); (C) predischarge chest X-ray on POD9 after administration of octreotide showed bilateral lung re-expansion and sharp bilateral costophrenic angles (arrows).

**Figure 2.**
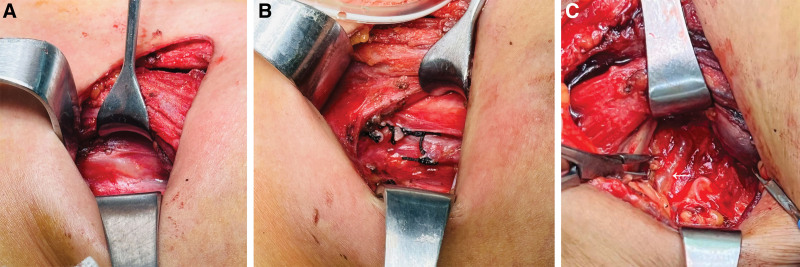
(A) The thoracic duct (TD) of case 1 was identified intraoperatively (arrow). (B) The TD of case 1 was ligated intraoperatively (arrow). (C) The TD of case 2 was identified intraoperatively (arrow).

**Figure 3.**
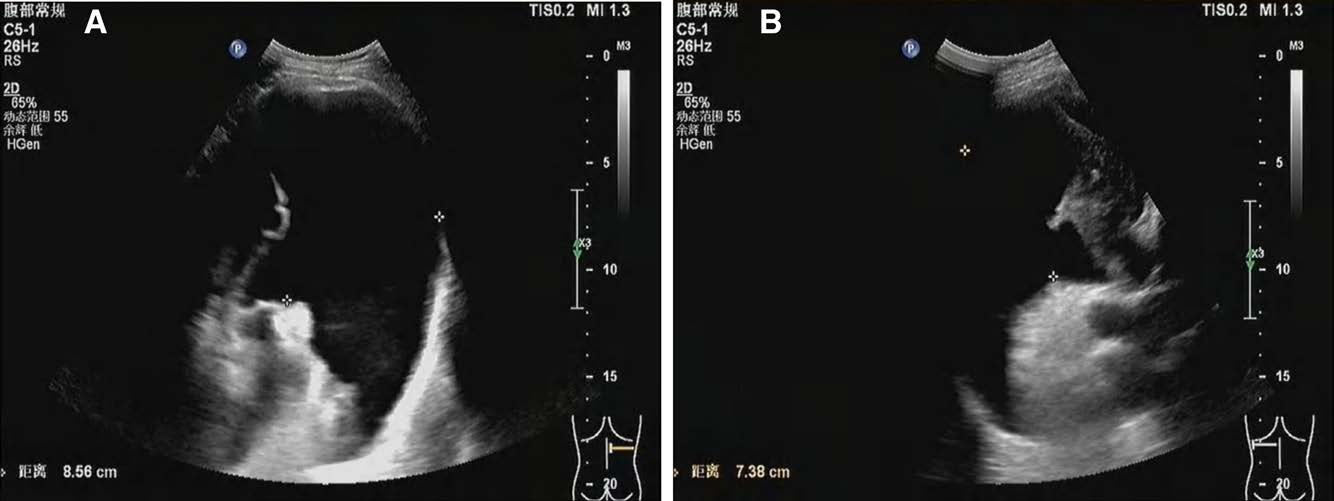
Ultrasound examination revealed bilateral pleural effusion: (A) right pleural cavity: massive effusion with a maximum depth of 8.6 cm (arrow). (B) Left pleural cavity: massive effusion with a maximum depth of 7.4 cm (arrow).

**Figure 4.**
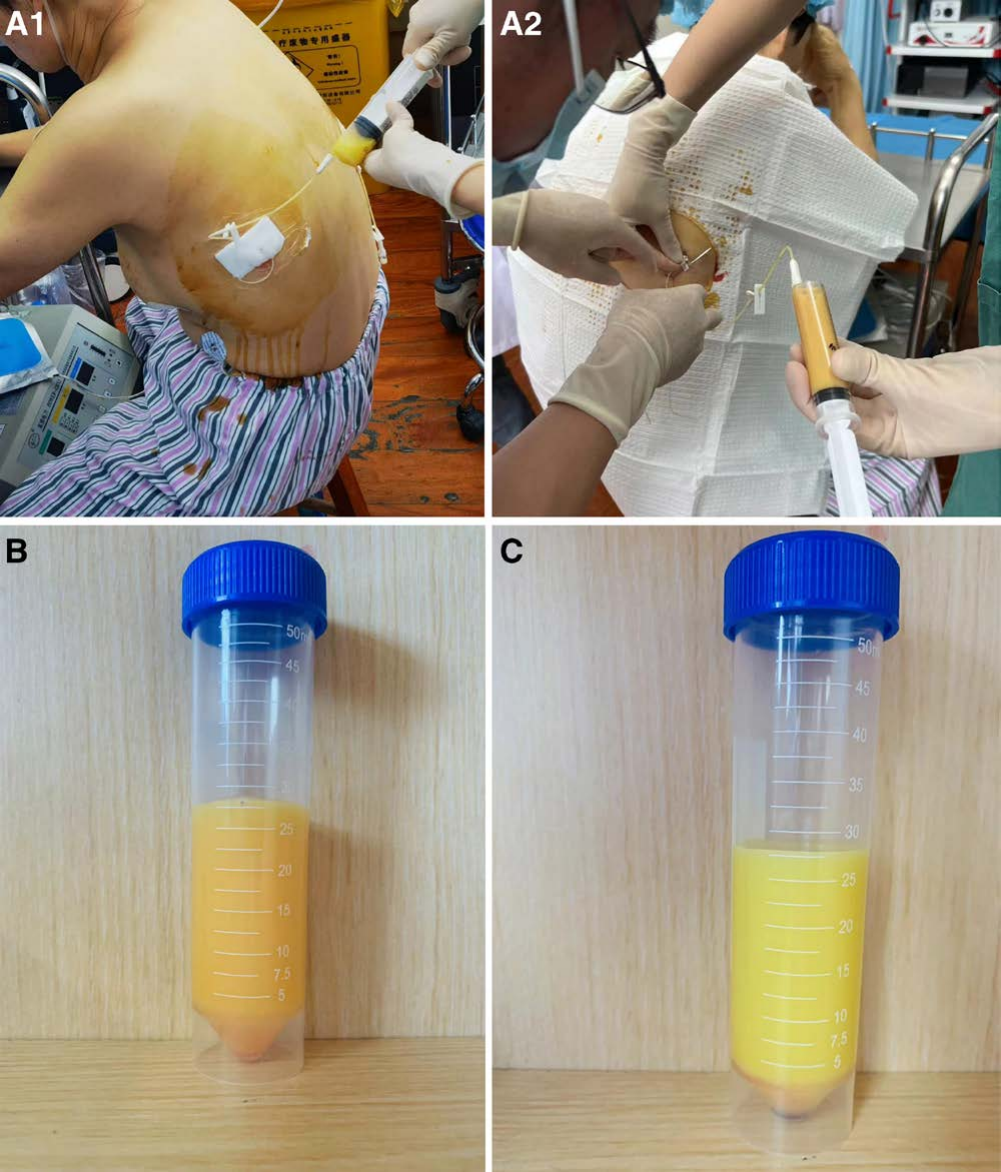
(A1) On POD4, right pleural cavity underwent thoracentesis for drainage. (A2) On POD4, left pleural cavity underwent thoracentesis for drainage. (B) The drainage fluid from the thoracentesis showed a chylous appearance. (C) After standing, the chylous fluid separated into layers.

**Figure 5.**
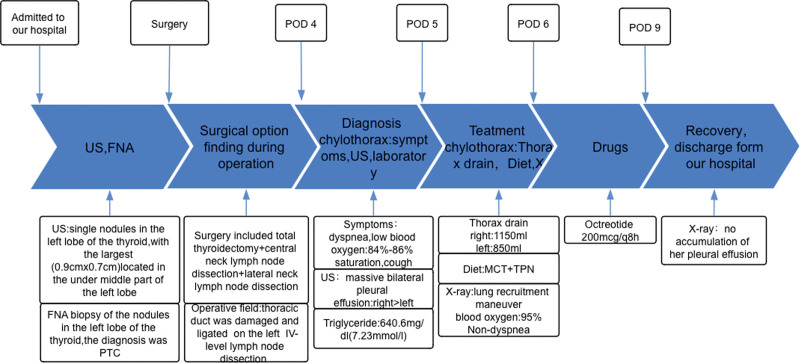
Flowchart of the management process for case 1.

A 56-year-old East Asian woman was diagnosed with PTC based on a fine needle aspiration biopsy of a 9 × 7 mm nodule in the lower left portion of the thyroid gland. She underwent surgery, specifically a total thyroidectomy with bilateral central lymph node dissection (BCLND) and left lateral lymph node dissection (LLND). The preoperative chest X-ray appeared normal (Fig. 1A). During the surgery, a frozen section analysis indicated the presence of a micro-PTC (0.5 cm) in the left thyroid lobe. There was no evidence of cancer metastasis in the prelaryngeal lymph node (0/1), but the pretracheal lymph nodes (2/2) and left paratracheal lymph nodes (1/2) were diagnosed with cancer metastasis. While dissecting the left level IV lymph nodes, a slight chyle leakage (CL) from the TD was observed (Fig. 2A). The TD was ligated 4 times (Fig. 2B). Lung inflation and a Valsalva maneuver were performed, confirming no further evidence of CL. The incision was closed, and drainage tubes were placed in the left lateral region and the central region. Postoperatively, the drainage output was normal, with a volume of 50 mL. However, on the 4th postoperative day (POD), the patient experienced dyspnea, and her oxygen saturation dropped to 84% to 86%. Physical examination revealed dullness on percussion and diminished and distant breath sounds bilaterally. There were no obvious signs of neck swelling, and the drainage tubes in the neck appeared normal. The left neck drainage tube had drained 10 mL of dark red fluid, while the right neck drainage tube had drained 25 mL of dark red fluid. Chest ultrasonography showed a large amount of fluid in both pleural cavities (Fig. 3). As a result, a thoracentesis was performed (Fig. 4), and pigtail catheters were inserted for drainage. Initially, 300 mL of chylous fluid was drained from the left side and 300 mL from the right side (Fig. 4A), confirming the diagnosis of BC. The fluid analysis revealed positive results for the chyle test (Fig. 4B), with the Livan test (+). The total number of cells was 7650 × 10^6^/L, the total number of leukocytes was 1650 × 10^6^/L, lactate dehydrogenase was 274 U/L, and triglyceride levels were 7.23 mmol/L. During the first 24 hours of medium-chain triglycerides + total parenteral nutrition (TPN) administration, 850 mL of chylous fluid was drained from the left side and 1150 mL from the right side. Follow-up chest X-ray showed lung re-expansion (Fig. 1B), relief of dyspnea, and oxygen saturation levels fluctuating around 95%. Over the next 24 hours, the color of the chylous fluid became lighter (Fig. 4C), and the drainage volume reduced to 350 mL on the left side and 650 mL on the right side. Subsequently, octreotide 200 mcg subcutaneous injections were administered every 8 hours. The drainage output was monitored (Table 1). On POD9, the chest and neck drainage tubes were removed, and a chest X-ray was performed (Fig. 1C). The patient was discharged from the hospital. The postoperative histopathological examination revealed a left PTC with a maximum diameter of approximately 0.5 cm. Lymph node metastases (LNM) were identified in the cervical lymph nodes (8/23). The treatment process is illustrated in Figure 5.

**Table 1:** The drainage of pleural effusion after the first 8 hours of octreotide injection.

	Left drainage fluid (mL)	Right drainage fluid (mL)	Color
The 1st 2 hours	15	33	Pale yellow and clear
The 2nd 2 hours	8.5	0	Pale yellow and clear
The 3rd 2 hours	9.2	0	Pale yellow and clear
The 4th 2 hours	7.2	0	Pale yellow and clear

### 2.2. Case 2

**Figure 6.**
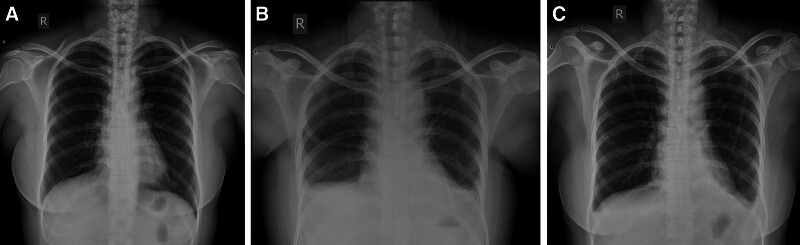
Chest X-rays of case 2: (A) preoperative chest X-ray was normal. (B) On postoperative day 1 (POD1), bilateral pleural effusion was observed: blunting of bilateral costophrenic angles. (C) Predischarge chest X-ray on POD9 after administration of octreotide showed lung re-expansion, sharp right costophrenic angle, and slightly blunted left costophrenic angle.

A 25-year-old East Asian female presented with a solid hypoechoic nodule measuring approximately 26 × 18 mm in the left lobe of the thyroid, which was diagnosed as PTC through fine needle aspiration. Simultaneously, LNM in the left level IV region was confirmed through lymph node biopsy. The preoperative chest X-ray was normal (Fig. 6A). The surgical procedure involved total thyroidectomy + BCLND + LLND. Intraoperatively, adhesions were observed between the left recurrent laryngeal nerve and left paratracheal lymph nodes, which were released and dissected. The left level IV lymph node was enlarged with a maximum diameter of approximately 2.5 cm. During the dissection of the left level IV lymph nodes, CL from the TD was observed and ligated (Fig. 2C). Lung inflation and Valsalva maneuver were performed without evidence of CL, and the incision was closed. One drainage tube was placed in the left lateral compartment, and another in the central compartment. On the POD1, the patient complained of chest distress and dyspnea. The neck drainage tubes were normal, and the drainage volume from both sides was approximately 160 mL/day. The chest X-ray showed that the left septal plane was not clear, bilateral rib diaphragm angle became obtuse, suggesting pleural effusion (Fig. 6B). Due to the patient’s young age and absence of special medical history, CL causing BC was considered. The patient was treated with TPN and octreotide 200 mcg administered every 8 hours for 6 days. On POD7, a follow-up chest X-ray showed gradual improvement (Fig. 6C), and the patient was gradually transitioned to a normal diet. The drainage tubes were removed, and the patient was discharged on POD10. Postoperative examination results showed a left PTC with a maximum diameter of approximately 1.8 cm. LNM were identified in the cervical lymph nodes (11/35).

## 3. Literature review

### 3.1. Methods

**Figure 7.**
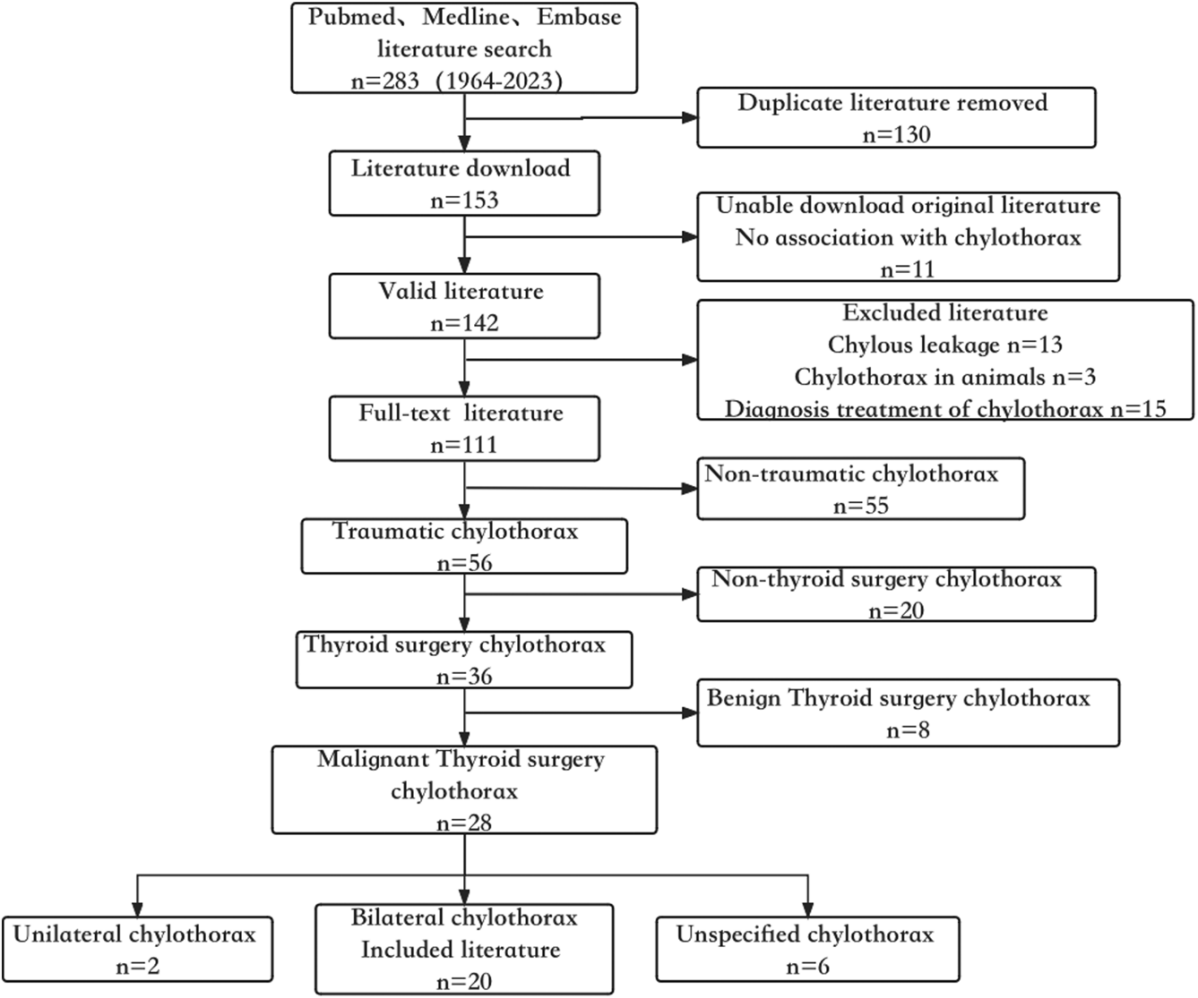
Flow diagram of the search and review process.

We conducted a comprehensive literature review on postoperative chylothorax after thyroid cancer, covering articles published from 1964 to 2023. The PubMed (69), Embase (145), and Medline (69) databases were searched using the keywords “Thyroid” and “chylothorax.” A total of 283 articles were retrieved and independently reviewed by 2 authors, Zhou Jing and Li daxue. Duplicate articles (130) were excluded, leaving 153 articles. Eleven articles were excluded due to the inability to access the original literature or low relevance without clear mention of chylothorax. After screening, 142 valid articles were included. Each of the included articles, as well as their references, was thoroughly studied and downloaded based on a comprehensive review of titles, abstracts, and full-text. Thirteen articles simply describing chylothorax, 3 articles on animal chylothorax, and 15 articles on chylothorax diagnosis and treatment methods were excluded. Finally, 111 full-text articles were downloaded. Among these, 55 articles on nontraumatic chylothorax were excluded, and among the 56 articles on traumatic chylothorax, 36 articles were related to chylothorax caused by thyroid surgery, including 8 articles on chylothorax caused by surgery for benign thyroid tumors, and 28 articles on chylothorax after thyroid malignancy surgery, including 2 articles on unilateral chylothorax and 6 articles that did not specify the side of chylothorax. Finally, 20 relevant articles were selected for inclusion in our review, comprising 18 case reports and 2 retrospective studies. For each included study, comprehensive manual data extraction was performed by 2 authors, focusing on key aspects such as surgical scope, diagnostic methods, treatment strategies, and management outcomes. The methods and data selection process used in our literature screening are illustrated in Figure 7.

### 3.2. Results

A total of 29 patients were included, with 27 from the previous literature and 2 from this case report. Table 2 and Table 3 provide a summary of the essential information, pathological type, surgical approach, timing of chylothorax occurrence, symptoms, diagnosis, treatment, and outcomes of the 29 patients. The average age of patients at the time of diagnosis was 44 years (range: 17–65 years), with a female-to-male ratio of 10:1. Chylothorax was more frequently observed in cases where the tumor was located on the left side, accounting for 65.4% (17 cases), while only 1 case was found on the right side, and 8 cases were bilateral. Among the 17 cases of left-sided thyroid cancer, except for 2 cases with an unknown extent of LND and 1 case without left LND, the remaining 14 patients underwent left LND. In the case of tumors located on the right side, cervical LND involved BCLND and left LND. Among the 8 cases with bilateral tumors, all underwent left LND (8 cases with bilateral LND, 1 case with left LND). Five patients underwent simultaneous superior mediastinal LND. Among the 24 patients with mention of TD, TD was found in 16 cases, injury in 14 cases, and intraoperative ligation was performed in 2 cases despite no TD injury.^[12,19]^ In addition to our 2 cases, hemostatic agents such as hemostatic gauze and fibrin glue were used for reinforcement during duct ligation, and no significant chylous leakage was observed intraoperatively. Among the 24 cases with mention of CL, 8 cases also had cervical CL, 5 cases were caused by intraoperative TD injury, and 3 cases had no apparent TD injury but still developed cervical CL.

**Table 2:** Study type, patients characteristics and type of surgery, symptoms, diagnostics, treatment and outcome of chylothora.

References/year	Cases	Age (years)	Gender	Tumor location	Pathological type	Area of LND			Management of TD			Cervical chyle leak	LNM	Postoperative symptoms	POD of chylothorax	More chylothorax (side)	Diagnosis		Treatment							Recovery time (days)
						CLND	LLND	SPLND	Identification	Injury	Ligation						Imaging (X; US; CT; lymphangiography)	Laboratory (triglyceride) mg/dL		Thorax drain (volume)		Thorax drain (days)	Diet (MCT; TPN; fat-free diet)	Drugs	Surgical treatment	
1. Almazan, E.P. (2023)^[8]^	1	61	Female	N/A	PTC	Bilat.	Bilat.	Yes	N/A	N/A	N/A	N/A	N/A	Dyspnea; low blood oxygen saturation; neck swelling	Immediately	Left	X; CT	R1327; L1563	1450		<1		Fat-free diet	Octreotide 100 mcg TID	No	N/A
2. Jeckowski, M.P. (2023)^[10]^	1	63	Female	N/A	MTC	Bilat.	Left	Yes	Yes	Yes	Yes	No	N/A	Chest pain; dyspnea	1	Right	X	Yes	2200		11		MCT; TPN	No	No	15
3. Shenoy, U. (2022)^[11]^	1	59	Female	N/A	PTC	Bilat.	No	N/A	No	No	No	No	N/A	Chest pain; dyspnea; low blood oxygen saturation	Immediately	Left	X; US	N/A	550		<1		TPN	N/A	No	32
4. Angeramo, C.A. (2021)^[3]^	1	30	Female	Left	PTC	Bilat.	Left	N/A	No	No	No	Yes	N/A	Chest pain	3	Left	X	795	Yes		<1		MCT	Octreotide 100 mcg/8 h	No	4
5. Kashoob, M. (2021)^[12]^	1	35	Female	Left	PTC	Bilat.	Left	N/A	Yes	Yes	Yes	No	11/88	Chest distress; dyspnea; low blood oxygen saturation	7	Left	X	505.02	2500		7		Fat-free diet	Octreotide 100 mcg/8 h	No	7
6. Lee, J. (2020)^[9]^	3	N/A	N/A	Left	PTC	Bilat.	Left	N/A	N/A	N/A	N/A	N/A	N/A	N/A	N/A	Left	X	N/A	N/A		N/A		Fat-free diet	No	No	N/A
		N/A	N/A	Left	PTC	Bilat.	N/A	N/A	N/A	N/A	N/A	N/A	N/A	N/A	N/A	Left	X	N/A	N/A		N/A		Fat-free diet	No	No	N/A
		N/A	N/A	Left	PTC	N/A	N/A	N/A	N/A	N/A	N/A	N/A	N/A	N/A	N/A	Left	X	N/A	N/A		N/A		Fat-free diet	No	No	N/A
7. Sharma, A. (2018)^[13]^	1	41	Female	Left	PTC	Left	No	N/A	No	No	No	No	1/5	Chest pain; dyspnea; cough	3	Left	X; lymphangiography	1996	Yes		N/A		Fat-free diet	No	Percutaneous embolization of the thoracic duct	N/A
8. Hayashibara, N. (2016)^[14]^	1	48	Female	Left	PTC	Bilat.	Left	N/A	No	No	No	No	4/22	Dyspnea	4	Left	X	N/A	1400		5		Fat-free Diet	Octreotide 50mcg/d	No	16
9. Merki, V. (2016)^[6]^	1	54	Male	Left	PTC	Bilat.	Left	N/A	Yes	Yes	Yes	Yes	N/A	Chest pain; dyspnea; low blood oxygen saturation	2	Right	CT	Yes	Yes		18		TPN; MCT	No	No	22
10. Runge, T. (2014)^[15]^	1	40	Female	Bilat.	PTC	Bilat.	Bilat.	N/A	Yes	No	Yes	No	Yes	Dyspnea	2	Same	CT	1506.2	4000		7		TPN	Octreotide	Yes	**9**
11. LI Zhi Yu. (2013)^[16]^	4	48	N/A	Left	PTC	Left	Left	N/A	Yes	Yes	Yes	Yes	N/A	Chest distress; dyspnea; chest discomfort	2	Same	X	Yes	Yes		Yes		TPN	No	No	6
		65	N/A	Left	PTC	Left	Left	N/A	Yes	Yes	Yes	Yes	N/A	Chest distress; dyspnea; chest discomfort	8	Same	X	Yes	Yes		Yes		TPN	No	No	4
		40	N/A	Bilat.	PTC	Bilat.	Bilat.	N/A	Yes	Yes	Yes	Yes	N/A	Chest distress; dyspnea; chest discomfort	2	Same	X	Yes	No		No		TPN	No	Yes	**17**
		31	N/A	Bilat.	PTC	Bilat.	Bilat.	N/A	Yes	Yes	Yes	YES	N/A	Chest distress; dyspnea; chest discomfort	3	Same	X	Yes	Yes		Yes		TPN	No	No	7
12. Tian Wei (2012)^[17]^	2	40	Female	Bilat.	PTC	Bilat.	Bilat.	N/A	Yes	Yes	Yes	No	N/A	Dyspnea; chest discomfort	2	Right	X	Yes	1340		17		TPN	Somatostatin/Octreotide	No	20
		31	Female	Bilat.	PTC	Bilat.	Bilat.	N/A	Yes	No	Yes	No	N/A	Dyspnea; chest discomfort	3	Right	X	Yes	940		7		TPN	Somatostatin/Octreotide	No	8
13. Tallon-Aguilar, L. (2010)^[18]^	1	38	Female	Left	PTC	Bilat.	Left	N/A	N/A	N/A	N/A	N/A	N/A	Dyspnea	3	Left	X	N/A	1000		N/A		TPN	No	Yes	N/A
14. Khurana, H. (2009)^[19]^	1	17	Female	Bilat.	PTC	Bilat.	Bilat.	Yes	No	No	No	Yes	N/A	Dyspnea; low blood oxygen saturation; neck swelling	Immediately	N/A	X	N/A	Yes		N/A		TPN	Octreotide 100 mcg q8 h	Yes	**12**
15. Han, C. (2009)^[20]^	1	42	Female	Bilat.	MTC	Bilat.	Bilat.	Yes	Yes	Yes	Yes	No	Yes	Chest pain; dyspnea	8	Left	X	283	3040		4		TPN	No	No	5
16. Roh, J. (2008)^[21]^	1	63	Male	Left	PTC	Left	Left	Yes	No	No	No	Yes	Yes	N/A	27	N/A	N/A	N/A	N/A		N/A		MCT; TPN	No	Yes	**35**
17. Bae, J. (2007)^[22]^	2	46	Female	Bilat.	PTC	Bilat.	Left	N/A	No	No	No	No	Yes	Dyspnea; chest discomfort	7	Left	X; CT	459	2800		N/A		TPN	No	No	15
		47	Female	Left	PTC	Bilat.	Left	N/A	No	No	No	No	Yes	Dyspnea; chest discomfort	3	Left	X; CT	959	700		3		TPN	No	No	6
18. Tsukahara, K. (2007)^[23]^	1	72	Female	Left	PTC	Bilat.	Left	N/A	Yes	Yes	Yes	No	Yes	Dyspnea	2	Right	X	N/A	1710		<1		TPN	No	No	6
19. Jabbar AS. (1995)^[24]^	1	47	Female	Right	PTC	Bilat.	Bilat.	N/A	Yes	Yes	Yes	No	Yes	Chest pain; dyspnea	4	Right	X	Yes	2000		<1		No	No	No	6
20. Har-EL, G. (1985)^[25]^	1	34	Female	Left	PTC	Bilat.	Left	N/A	Yes	Yes	Yes	No	N/A	Chest pain; dyspnea; low blood oxygen saturation	3	Right	X	N/A	160		<1		MCT	No	No	6
Present case	2	56	Female	Left	PTC	Bilat.	Left	N/A	Yes	Yes	Yes	No	8/23	Dyspnea; low blood oxygen saturation; cough	4	Right	X; US	640.6	3090		9		MCT; TPN	Octreotide 200 mcg q8 h	No	5
		25	Female	Left	PTC	Bilat.	Left	N/A	Yes	Yes	Yes	No		Chest distress; dyspnea; low blood oxygen saturation	1	Same	X	No	No		No		MCT; TPN	Octreotide 200 mcg q8 h	No	9

LLND = left lateral lymph node, LND = lymph node dissection, LNM = lymph node metastases, MCT = medium-chain triglycerides, POD = postoperative day, PTC = papillary thyroid carcinoma, TD = thoracic duct, TPN = total parenteral nutrition.

**Table 3:** Summary of the basic information, clinical Symptoms, diagnosis, and treatment of the 29 patients.

Characteristics	Number of cases (29)	Mean/percentage/range
Age (years)	26	44 (17–65)
Gender	22	
Female	20	90.9%
Male	2	9.1%
Tumor location	26	
Left	17	65.4%
Right	1	3.8%
Bilateral	8	30.8%
Area of LND		
CLND 28/96.6%	Bliat. 24/left 4	Bliat. 85.7%/left 14.3%
LLND 27/93.1%	Bliat. 9/left 16/No. 2	Bliat. 33.3%/left 59.3%/No. 7.4%
SPLND	5	17.2%
Pathological type	29	
PTC	27	93.1%
MTC	2	6.9%
Management of TD	24	
ID	16	66.7%
Injury	14	58.3%
LIG	16	66.7%
Cervical chyle leak 24	8	33.3%
Postoperative symptoms	25	
Chest distress	6	24.0%
Chest pain	8	32.0%
Dyspnea	24	96.0%
Chest discomfort	8	32.0%
Low blood oxygen saturation	8	32.0%
Neck swelling	2	8.0%
Cough	2	8.0%
POD of chylothorax	4	Immediately-27
More chylothorax (side)	27	
R > L	8	29.6%
L > R	13	48.1%
The same	6	22.2%
Diagnosis		
Imaging 28	28	100%
Laboratory (triglyceride) (mg/dL)	18	1003.4
Conservative treatment	29	100%
Thorax drain	23	79.3%
Thorax drain (volume: mL/days)		1805 (160–>4000)/5.9 (1–18)
Diet	28	96.6%
Drugs (octreotide)	10	34.5% (50 mcg/d-200 mcg/q8 h)
Surgical treatment	5	17.2%
Recovery time (days)	23	11.8 (4–35)
Conservative treatment (days)	19	10.5 (4–32)
Surgical treatment (days)	4	18.3 (9–35)

LLND = left lateral lymph node, LND = lymph node dissection, POD = postoperative day, PTC = papillary thyroid carcinoma, TD = thoracic duct, TPN = total parenteral nutrition.

The most common clinical symptoms of chylothorax were dyspnea (96.0%), followed by chest pain (32.0%), chest discomfort (32%), and chest distress (24%); neck swelling and cough were rare (8.0%). The average time from surgery to chylothorax diagnosis was 4 days (range: immediately to 27 days). Three patients experienced dyspnea immediately after surgery. A careful examination of the original literature revealed that bilateral celiac chest effusion was predominantly greater on the left side than the right side. Among the 27 cases, 13 cases (48.1%) had a larger amount of pleural effusion on the left side compared to the right side, and among these 13 cases, 9 cases had tumors located on the left side. In 7 cases, the surgical approach involved BCLND and left LLND without right LLND, only 2 of which were found to have TD injury and were ligated intraoperatively. Among the 27 cases, 8 cases (29.6%) had a larger amount of pleural effusion in the right pleural cavity than the left, and 6 cases (22.2%) had an equal volume of pleural effusion in both pleural cavities. TD were observed intraoperatively in these 14 patients, and in all 12 cases of injury, they were ligated.

All patients underwent imaging examinations, including standard chest X-ray, CT scan, and ultrasound, which revealed the presence of pleural effusion. Among the 18 cases that underwent laboratory examinations, quantitative measurement of triglycerides confirmed the diagnosis of chylothorax, with an average triglyceride value of 1003.4 mg/mL. Conservative treatment was successful in 24 out of 29 patients, with a success rate of 82.8%. Three cases were managed successfully through medication and dietary control alone. In 1 case,^[13]^ lymphangiography performed on POD 3 did not show any leakage from the TD and was successfully managed conservatively without repair. Among the 25 patients who underwent thoracentesis, 23 of them received thoracentesis drainage. The average drainage volume was approximately 1805 mL, and the average drainage duration was 5.9 days (range: 1–18 days). Ten patients received octreotide infusion at dosages ranging from 50 mcg/day to 200 mcg every 8 hours. Five patients who did not respond to conservative treatment required surgical intervention. Among these cases, 2 underwent cervical exploration on POD 4 [13], and case^[19]^ underwent exploration on POD3. In these cases, no apparent TD injury or leakage was found. The 3rd patient in case^[16]^ at POD3 to 4, case^[18]^ at POD 3 to 4, case^[19]^ at POD3, case^[20]^ at POD 4, and case^[21]^ at POD27 had unsuccessful conservative treatment. The average duration of drainage was 1 to 18 days. In case,^[21]^ after unsuccessful conservative treatment for 27 days, surgery was performed, which involved ligation of the fistula with gelatin sponge packing and transfer of the pectoralis major muscle flap to repair the TD. This case had the longest hospital stay, reaching 62 days. Among the 29 patients, 23 cases reported the recovery time, which was calculated as the time from the discovery of chylothorax to discharge. The average recovery time was 11.8 days. The average recovery time for conservative treatment was 10.5 days, while the average recovery time for surgical treatment was 18.3 days.

## 4. Discussion

### 4.1. Background

Differentiated thyroid cancer has a favorable prognosis, but it is associated with a high incidence of regional LNM. Complete LND contributes to improved patient prognosis. However, LND, particularly LLND, can lead to postoperative complications. Lymphatic leakage following cervical LND is a rare but potentially life-threatening complication, with an incidence ranging from approximately 4.5% to 8.3%.^[26–29]^ Another complication is chylothorax, which occurs in approximately 2% to 8% of cases following cervical LND.^[3,10,12]^ The incidence of BC is even lower, with literature reports indicating a rate of <1.85%.^[6,12,13,16]^ BC can cause extensive pleural effusion, leading to decreased oxygen saturation, respiratory distress, and even respiratory failure.^[26,28]^ Additionally, the significant loss of chyle fluid impairs immune function, causes nutritional depletion, delays wound healing, and increases the risk of wound infection, thereby prolonging hospitalization. Although rare, BC is a serious complication. To date, only 21 articles (including the present study) have reported on this topic.

### 4.2. Anatomical basis

The mechanism of BC following cervical LND in differentiated thyroid cancer is still a matter of debate. From an anatomical perspective, the TD is the largest lymphatic vessel in the human body, responsible for draining approximately 75% of the lymphatic fluid. It is a thin-walled tube composed of a single layer of cells, with a diameter ranging from 2 to 6 mm. The TD originates from the chyle pool at the level of the second or third lumbar vertebra. It enters the thoracic cavity through the aortic hiatus of the diaphragm and ascends in the posterior mediastinum, situated between the left descending aorta and the right brachiocephalic vein. At the level of the fifth thoracic vertebra, the TD tilts to the left and enters the superior mediastinum, passing posterior to the aortic arch and the left subclavian artery, between the esophagus on the left and the left pleura. Finally, the TD terminates at the confluence of the left subclavian vein and the internal jugular vein.^[7,30,31]^ However, the TD may also drain into nearby major veins near this confluence or give rise to several small lymphatic vessels before termination. Therefore, during left level IV LND, due to anatomical variations and the thin-walled structure of the TD, it is susceptible to injury or disruption, providing the anatomical basis for the development of chylothorax.

There are currently 2 hypotheses concerning the mechanism of BC following cervical LND for PTC: the “overflow hypothesis” and the “blocked drainage hypothesis.” The “overflow hypothesis” suggests that traumatic injury to the TD results in direct leakage into the mediastinum. This TD injury causes impregnation of the mediastinal tissues and an inflammatory reaction, leading to chyle flowing directly into the pleural cavity. On the other hand, the “blocked drainage hypothesis” proposes that ligation of the TD increases retrograde hydrostatic pressure, leading to elevated pressure within the TD and resulting in nontraumatic CL. Nontraumatic CL can infiltrate either the unilateral or bilateral pleural cavities, ultimately leading to chylothorax.^[6,9]^ Different experts in the literature hold different opinions on this matter. Some authors support the “overflow hypothesis,”^[6,11,13,14]^ while others propose the “blocked drainage hypothesis.”^[12,15–17]^ Some authors suggest that both mechanisms may coexist and influence each other.^[3,9,20,25]^

In the present case, the 2 patients are similar to the studies mentioned in the literature,^[22–24]^ where the TD was identified during surgery, and no cervical CL was observed intraoperatively or postoperatively. The possibility of chyle fluid leaking into the mediastinum due to TD injury is not considered, and greater consideration is given to increased TD pressure following ligation. Since the patients did not develop chylothorax immediately after surgery, it indicates that the accumulation of pleural effusion in the pleural cavity is a gradual process. The prompt improvement of symptoms in the patients with the use of TPN and octreotide, which reduces the source of chyle fluid, and the absence of further leakage thereafter, support the belief that the “blocked drainage hypothesis” better explains the development of BC in this case.

### 4.3. Reason for BC

The location of the thyroid tumor does not influence the occurrence of chylothorax. However, tumors located on the left side of the thyroid are more likely to involve the left side of LLNM. Extensive LLND, particularly involving the left level IV, plays a significant role in the development of chylothorax. In this study, among the 29 analyzed cases, 25 patients underwent left level IV LLND, creating anatomical conditions favorable for chylothorax, which is consistent with previous findings.^[6]^ Additionally, LLND in the upper mediastinum can also lead to TD injury.^[8,10,19–21]^ In 66.7% of the cases, the TD was ligated, and in 2 patients, ligation was performed even in the absence of injury. Similar to other cases,^[12,20]^ fibrin glue and hemostatic sponge were used in this study to reinforce TD repair, and no cervical CL was observed intraoperatively or postoperatively. Therefore, it can be inferred that CL caused by complete ligation of the TD after injury may occur in the thoracic region rather than the cervical region. This is especially true when careful repair, ligation, and the use of hemostatic materials are employed. For instance, in a study,^[13]^ lymphangiography for TD imaging showed no obvious leakage or damage to the TD, but a significant expansion of TD branches below the cervical hemostatic clip was observed. This indicates that excessive pressure on the cervical region after TD repair, complete detachment of the TD as described in,^[24]^ or the use of cervical hemostatic clips or other hemostatic materials and ligation during surgery can result in complete ligation of the TD, preventing chyle from flowing back into the veins.^[13]^ These observations align with the principle of the “blocked drainage hypothesis” as the underlying cause of BC.

Among the 24 cases that mentioned CL, only 8 cases actually experienced CL, resulting in a low incidence rate. Out of these 8 cases, only 5 cases had confirmed TD injury. This is because in some cases, the TD was not completely damaged, and complete ligation was not performed, which resulted in CL from the cervical region. However, due to excessive chyle accumulation and poor drainage in the cervical region, the chyle leaked into the mediastinum, leading to the development of chylothorax. These patients generally had concurrent cervical CL.^[6,16,17]^ Therefore, it is crucial to identify the TD, especially at the jugular vein angle, when performing LND in the left level IV. If the TD is identified, any leakage should be promptly examined, and efforts should be made to avoid detachment, complete ligation, and excessive compression. The volume of bilateral pleural effusion in BC varied among cases, with the left side typically having a greater volume than the right side. Among patients with this characteristic, 48.1% had a larger volume of left-sided pleural effusion compared to the right side. Among these patients, 69.2% had tumors located on the left side, and 53.8% underwent BCLND + left LLND surgery without right LLND. Among them, only 2 cases had intraoperative TD injury observed and ligation performed.^[12,20]^ This may be attributed to the course of the TD in the thoracic cavity. The main course of the TD in the upper mediastinum is typically on the left side. Therefore, when mediastinal CL occurs, chyle is more likely to enter the left pleural cavity, which explains why chylothorax is more commonly observed on the left side and why the left pleural cavity tends to have a larger volume in BC.^[7,30]^

An astonishing finding in the study is that nearly one-third of patients with BC had a greater volume of pleural effusion on the right side. Further analysis of the literature did not reveal any significant differences in the surgical approach or tumor location among these cases.^[6,10,17]^ Additionally, 22.2% of the patients had an equal amount of pleural effusion bilaterally, and there were no apparent differences in the surgical approach or tumor location in these cases either.^[15,16]^ Statistical analysis showed that all 14 of these patients had the TD identified intraoperatively, and among them, 12 cases had TD injury, which were subsequently ligated. Due to intraoperative injury, chyle fluid leaked into the mediastinum prematurely. In addition to the leakage caused by increased hydrostatic pressure within the ligated TD, chyle that entered the pleural cavity also included chyle that leaked into the mediastinum as a result of intraoperative TD injury.

### 4.4. Diagnosis

Diagnosing chylothorax after thyroid cancer surgery can be challenging as it is often overlooked and not easily detected at an early stage. The primary symptom of chylothorax is typically dyspnea, which gradually worsens over time (96.0% of cases). The average time from surgery to chylothorax diagnosis is 4 days, which is related to the gradual leakage of chyle into the pleural cavity and the resulting compression of lung tissues, leading to dyspnea. Severe hypoxemia is commonly associated with the rapid accumulation of a large amount of chyle fluid in the pleural cavity. Among the 29 patients in this review, 3 patients experienced immediate dyspnea after surgery,^[8,11,19]^ and all showed significant decreases in oxygen saturation. Patient^[8]^ required the use of high-flow nasal cannula with a flow rate of 30 L and 50% FiO_2_. Patient^[11]^ had an oxygen saturation of 91% to 93%, and patient^[19]^ experienced severe hypoxemia with an oxygen saturation of 75% to 80%. Although no apparent TD injury was observed intraoperatively in these 3 cases, the immediate CL after surgery was still considered to be related to TD injury. This was likely due to the failure to detect the injury intraoperatively and repair it in a timely manner, resulting in substantial CL and poor cervical drainage, leading to severe CL.

In addition to dyspnea and other clinical symptoms, the diagnosis of chylothorax can be supported by imaging and laboratory examinations. Standard chest X-ray, CT scan, or ultrasound can confirm the presence of a significant amount of pleural effusion. Diagnostic thoracentesis can be performed to examine the appearance of the pleural fluid, which typically appears milky or chylous. Like the images presented in case 1 of this article, it is consistent with.^[3,8]^ In a few cases, there is a faint yellow serous consistency due to fasting.^[17,20]^

Laboratory analysis of the drained pleural fluid includes measuring triglyceride levels (>110 mg/dL or 1.2 mmol/L) and identifying fat globules under the microscope (chyle test), which can confirm the diagnosis of chylothorax. The chyle test has a diagnostic sensitivity of up to 100.0%, but its specificity is only 4.0%. Therefore, a positive chyle test alone cannot be used as a definitive diagnostic criterion for postoperative chylothorax.^[8,13,15]^ Lymphangiography is considered the “gold standard” for diagnosing chylothorax, but its use is limited due to the complex and invasive nature of the procedure.^[13,32]^

### 4.5. Treatment

There is currently no standardized treatment protocol for chylothorax following thyroid cancer surgery. The available treatment options include conservative treatment, surgical intervention, and interventional therapy. The choice of treatment should be based on a comprehensive assessment of the individual patient’s condition.

Conservative treatment is the initial approach for confirmed cases of chylothorax following thyroid cancer surgery. It serves as a preparatory step for other treatment options if necessary. The goal of conservative treatment is to reduce the formation of chyle and promote natural healing of the damaged TD.^[33]^ Recommended strategies include dietary modifications such as a high-protein, low-fat, medium-chain triglyceride diet, or TPN with fasting. Correcting water, electrolyte, and nutritional imbalances is also crucial. Octreotide has a certain therapeutic effect on chylothorax. Octreotide, a long-acting somatostatin, reduces visceral blood flow and lowers the triglyceride content in chyle fluid, providing the possibility of spontaneous healing of the fistula. Administration for 2 to 3 days can be effective for mild to moderate chylothorax.^[14]^ The success rate of using octreotide to treat post-esophagectomy chylothorax ranges from 38% to 100%.^[34]^ In the first case, the initial patient diagnosed with chylothorax began with dietary control; however, there was still a significant amount of pleural drainage. After using octreotide, there was a sudden decrease in the drainage fluid, ultimately leading to a successful conservative treatment. In the second case, the patient initiated early administration of octreotide in conjunction with a straightforward dietary control strategy. This combined conservative treatment approach resulted in a favorable outcome. Within conservative treatment, timely diagnosis and therapeutic procedures such as thoracentesis and chest tube drainage are crucial. These interventions help alleviate respiratory symptoms and play a significant role in successful conservative management. In this study, out of 29 patients who underwent conservative treatment, 79.3% achieved successful outcomes. Among the 23 patients treated conservatively, 19 of them underwent chest tube drainage, effectively alleviating symptoms such as respiratory distress in a timely manner. This approach facilitated lung re-expansion and helped prevent serious complications.

Surgical treatment: patients unresponsive to conservative treatment may require reexploration and repair of the original incision, along with TD ligation. The optimal timing for surgery lacks a standardized guideline. The average recovery time for the 19 conservatively treated patients in this study was 10.5 days, consistent with the literature.^[35]^ Surgical intervention should be considered if chyle volume exceeds 10 mL/(kg day) for 48 to 72 hours or persists for more than 11 days following conservative treatment. Additionally, surgical treatment is warranted in the presence of severe nutritional, metabolic disturbances, or immunosuppression. Among the 29 patients analyzed in this study, 5 cases were unresponsive to conservative treatment and necessitated surgical intervention. Two patients underwent exploration, revealing no apparent TD damage or leakage. In case^[13]^ lymphangiography did not detect any significant TD injury-related leakage, with continued conservative treatment, the chylothorax resolved. The underlying mechanism for post-thyroid cancer surgery chylothorax formation tends to support the “drainage tube obstruction hypothesis.” Therefore, it is suggested that patients who have clearly observed the TD during surgery, undergone ligation, and experienced no significant postoperative cervical CL, with symptoms appearing later rather than immediately after surgery, are more suitable for conservative treatment. This approach may be supplemented with medications such as octreotide, while closely monitoring chyle fluid drainage from the pleural cavity.

Interventional treatment: since the first report by COPE^[36]^ on percutaneous embolization of the TD guided by lymphangiography for the treatment of chylothorax, TD embolization has rapidly emerged as a promising technique in recent years. It has also been utilized in patients with chylothorax following thyroid cancer surgery,^[37,38]^ demonstrating high success rates. In cases where conservative treatment fails after surgical intervention, interventional embolization can be performed to identify and occlude the TD fistula. Even if embolization is unsuccessful, it can provide valuable guidance for subsequent surgical interventions.^[39]^ However, this procedure demands a high level of expertise, particularly in cases involving anatomical variations of the TD, which adds to its complexity. The success and reproducibility rates of TD embolization vary among different medical centers, and its widespread adoption is still limited.

## 5. Conclusion

The underlying mechanism of BC is more inclined towards the “obstruction of the drainage ducts” hypothesis, especially when performing left level IV LND for thyroid cancer, which can result in injury to the TD. In order to prevent leakage, surgeons may completely ligate or transect the TD and apply tight pressure dressings, leading to excessive pressure within the TD in the mediastinum and nontraumatic leakage of chyle into both pleural cavities. During cervical LLND for differentiated thyroid cancer, it is crucial to identify the lymphatic vessels, particularly during left-sided LLND. If injury to the TD is encountered during surgery, immediate repair and ligation should be performed. However, complete ligation or interruption of the TD should be avoided to prevent CL. It is important to exercise caution to avoid excessive pressure on the cervical accessories. After identifying the TD or TD injury with CL during surgery, close monitoring of the patient’s respiratory condition is necessary. Postoperatively, regular chest X-ray follow-up should be conducted. In the presence of chylothorax, conservative treatment should be initiated for cases with minimal effusion, including dietary control and medication. If there is a moderate to large amount of effusion, chest tube drainage should be considered, and surgical intervention should be considered if chyle volume exceeds 10 mL/(kg day) for 48 to 72 hours or persists for more than 11 days following conservative treatment. Additionally, surgical treatment is warranted in the presence of severe nutritional, metabolic disturbances, or immunosuppression.

## Acknowledgments

The authors would like to thank the First Affiliated Hospital of Chongqing Medical University and the Women and Children’s Hospital of Chongqing Medical University for their support.

## Author contributions

**Conceptualization:** Qian Xiao, Ting Yang, Han Gao.

**Data curation:** Daxue Li, Qian Xiao, Ting Yang.

**Formal analysis:** Daxue Li, Qian Xiao.

**Funding acquisition:** Yuchen Zhuang.

**Investigation:** Daxue Li, Yuchen Zhuang, Ting Yang.

**Methodology:** Yuchen Zhuang, Ting Yang.

**Project administration:** Yuchen Zhuang.

**Resources:** Jing Zhou, Song Xue.

**Software:** Yuchen Zhuang, Ting Yang.

**Supervision:** Jing Zhou, Daxue Li, Ting Yang.

**Validation:** Song Xue.

**Writing – original draft:** Jing Zhou, Daxue Li, Han Gao.

**Writing – review & editing:** Xinliang Su, Han Gao.
